# The relationship between transition shock and professional identity of nursing interns in China: a moderated mediation effect analysis

**DOI:** 10.3389/fpsyg.2026.1886969

**Published:** 2026-08-04

**Authors:** Sai Liang, Naifu Tang, Han Ban, Mingyu Xue, Yanru Qin, Weiling Shi, Yongyi Chen

**Affiliations:** 1 Department of Oncology, The First Affiliated Hospital of Zhengzhou University, Zhengzhou, Henan, China; 2 Department of Emergency, The First Affiliated Hospital of Zhengzhou University, Zhengzhou, Henan, China; 3 Department of Nursing, The Affiliated Tumor Hospital of Xiangya School of Medicine, Central South University, Changsha, Hunan, China

**Keywords:** feedback-seeking behavior, nursing interns, professional identity, self-efficacy, transition shock

## Abstract

**Background:**

Nursing interns often experience transition shock upon entering clinical practice, which may diminish their professional identity and potentially lead to future turnover. Self-efficacy and feedback-seeking behavior are positive psychological resources for coping with occupational stressors and may be associated with a stronger sense of professional identity. This study aimed to investigate the relationship between transition shock and professional identity among nursing interns and to examine the mediating role of feedback-seeking behavior and the moderating role of self-efficacy in this process.

**Methods:**

A cross-sectional survey was conducted among 450 clinical nursing interns in China using an online questionnaire. The survey comprised the Transition Shock Scale for Undergraduate Nursing Students, the General Self-efficacy Scale, the Feedback-seeking Behavior Scale, and the Professional Identity Questionnaire for Nurse Students. The R package “mediation” and the bootstrap method were used to examine the mediating effect. The R package “lavaan” was further used to test the moderated mediation model. A two-sided *p*-value < 0.05 was considered statistically significant.

**Results:**

Transition shock was negatively correlated with professional identity (*r* = −0.754, *p* < 0.001). Feedback-seeking behavior was negatively correlated with transition shock (*r* = −0.711, *p* < 0.001) and positively correlated with professional identity (*r* = 0.862, *p* < 0.001). Feedback-seeking behavior partially mediated the relationship between transition shock and professional identity (indirect effect = −0.473, 95% CI: −0.557 to −0.391), with the indirect effect accounting for 56.3% of the total effect. Self-efficacy significantly moderated the first stage of the mediation pathway (transition shock to feedback-seeking behavior; β = 0.042, *p* < 0.001), thereby buffering the negative impact of transition shock on feedback-seeking behavior.

**Conclusion:**

Transition shock has a direct negative association with nursing interns’ professional identity, and feedback-seeking behavior acts as a partial mediator in this relationship. Self-efficacy plays a significant moderating role by buffering the negative association of transition shock with feedback-seeking behavior. These findings provide an empirical basis for hospitals and nursing schools to develop effective strategies aimed at mitigating the impact of transition shock, thereby potentially enhancing professional identity and contributing to the stabilization of the nursing workforce.

## 1 Introduction

Professional identity refers to an individual’s positive perception and affirmation of their occupation, and it serves as an important psychological foundation for individuals to complete work tasks and achieve organizational goals (Wu et al., 2016; Zhu et al., 2024). Nurse professional identity refers to the nurse’s recognition of the value of the nursing profession and the corresponding behaviors they demonstrate based on that recognition (Duan et al., 2017; Geoghan Marold et al., 2025). The professional identity among nurses is associated with their behavior in clinical work, which in turn results in varying levels of nursing quality (Wang T. T. et al., 2024; Zhou et al., 2019). Gong et al. (2025) found that lower professional identity is connected with career decision failures or erroneous decisions, severely lowering the quality of nursing practice. Nursing interns are an important part of future nursing workforce, and their employment quality is related to the development of nursing practice (Geng et al., 2026; Shang et al., 2021). The clinical internship stage is a key phase for nursing students to form their professional values, make career choices, and successfully transition into nursing. Therefore, exploring the professional identity of nursing interns and its associated mechanism is of significance.

Transition shock refers to the negative experiences such as doubt, confusion, bewilderment, that an individual faces when transitioning from a known role to an unfamiliar one, due to changes in knowledge, relationships, responsibilities, etc. (Duchscher, 2009; Xin et al., 2024). A cross-sectional study by Zhao et al. (2024) involving 387 nursing interns found that the transition shock experienced by nursing students during the clinical internship phase due to the dual changes in role and environment can reduce their professional identity, which may subsequently lead them to abandon the nursing profession. The transition shock theory proposed by Wang M. et al. (2024) suggests that the career transition shock experienced by individuals can lead them to question their previously formed professional identity (Duchscher, 2008; Li et al., 2024).

Transition shock is an important part of students’ clinical learning experience (Ding et al., 2024). It can be inferred that the transition shock experienced by nursing interns may influence their professional identity, but the relationship between the two and the mechanisms of their interaction still need further exploration. In-depth exploration of the intrinsic mechanisms and boundary conditions of how transition shock is associated with professional identity is of significant theoretical and practical importance for formulating effective intervention strategies and stabilizing the nursing workforce.

Based on Social Cognitive Theory, an individual’s cognitive and behavioral responses in stressful environments are the core factors influencing their psychological adaptation outcomes (Lu et al., 2026; Smoktunowicz et al., 2017). Feedback-seeking behavior (FSB), as an active and strategic social interaction behavior, is a key way for interns to cope with uncertainty, acquire knowledge, calibrate self-awareness, and enhance clinical skills (Liang et al., 2025; McGinness et al., 2020). High-level transition shock may suppress interns’ willingness to actively seek feedback, due to self-doubt or fear of exposing shortcomings. Feedback-seeking behavior is regarded as a positive learning strategy that helps individuals identify their shortcomings, adjust their behavior, and enhance their professional skills (Gong and Li, 2019; Wilson et al., 2024). In clinical environments, feedback-seeking behavior has been confirmed to be closely related to higher professional identity (Song et al., 2024). Thus, we speculate that feedback-seeking behavior may play a mediating role between transition shock and professional identity.

However, this mediating process may not be consistent for all individuals, and it is equally important to explore the boundary conditions of its effects. Self-efficacy, as an individual’s belief in their ability to successfully complete a certain task, is an important psychological resource in coping with stress and challenges (Berdida et al., 2023; Schwarzer et al., 1997). According to the “stress-resilience” framework, individuals with high self-efficacy are more likely to view transition shock as manageable challenges rather than threats, thereby maintaining the motivation and behavior to proactively seek feedback (Li et al., 2024; Tannenbaum et al., 1991). Conversely, interns with low self-efficacy may be more likely to adopt avoidance strategies when facing high pressure. Therefore, we hypothesize that self-efficacy may moderate the first half of the mediating pathway “transition shock → feedback-seeking behavior → professional identity,” specifically buffering the negative association between transition shock and feedback-seeking behavior.

**FIGURE 1:**
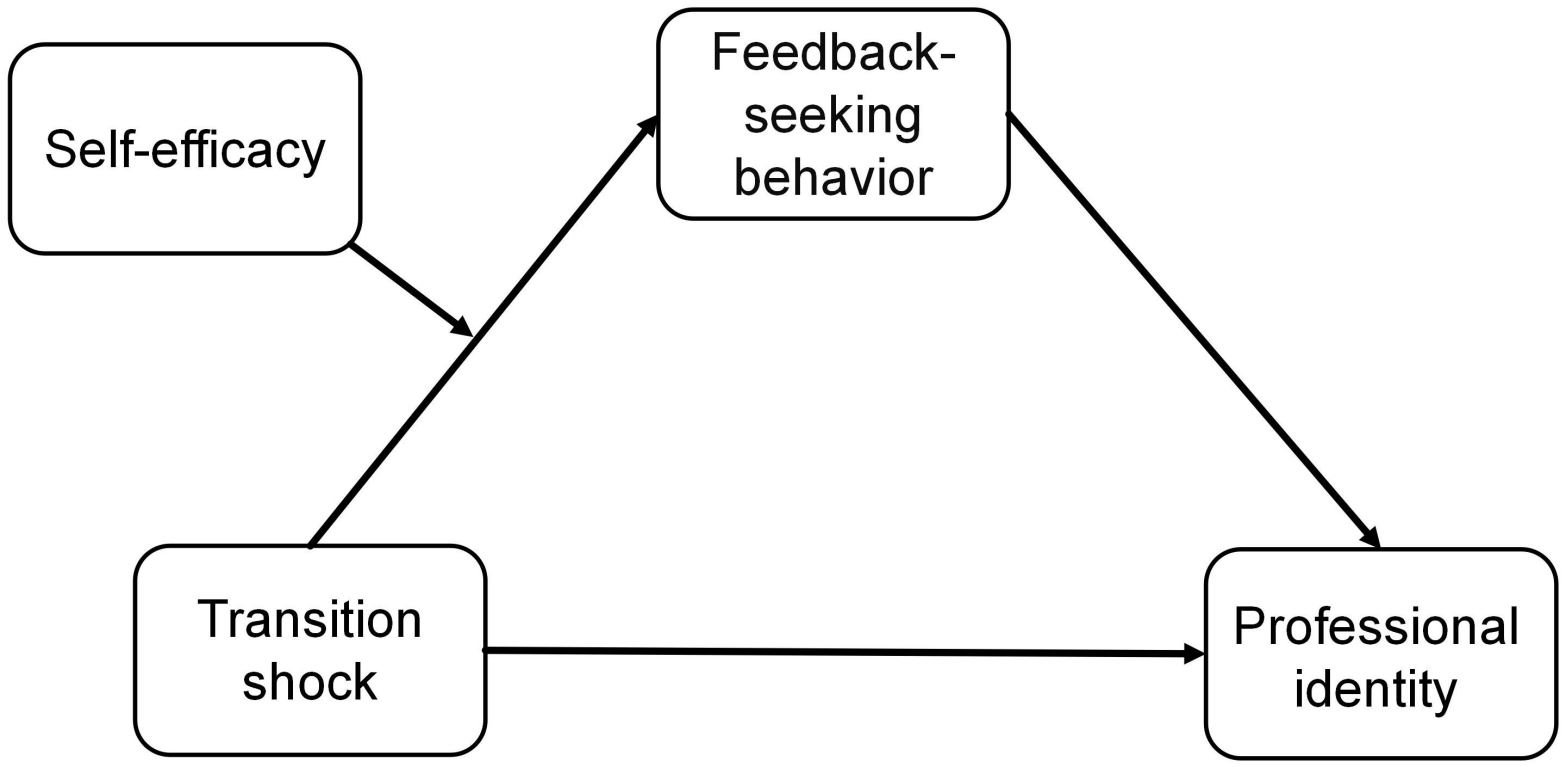
Hypothesized conceptual model.

In summary, self-efficacy and feedback-seeking behavior may play moderating and mediating roles, respectively, between transition shock and professional identity. This study aims to investigate the relationship between transition shock and nursing interns’ professional identity, and to examine the mechanisms of self-efficacy and feedback-seeking behavior in this process. The following hypotheses are proposed (Figure 1):

*H1*: Transition shock of nursing interns is negatively associated with professional identity.

*H2*: Feedback-seeking behavior and self-efficacy are positively associated with professional identity.

*H3*: Feedback-seeking behavior mediates the relationship between transition shock and professional identity.

*H4*: Self-efficacy moderates the path of “transition shock → feedback-seeking behavior” and the mediation model.

## 2 Materials and methods

### 2.1 Research design

From February 18 to March 19 in 2025, a cross-sectional study was conducted in China. This article is reported according to the STROBE guidelines (Cuschieri, 2019; Dewidar et al., 2025).

### 2.2 Sample

The target audience is clinical nursing interns cross China. A snowball sampling method was employed. Initially, the research team contacted clinical instructors in a tertiary hospital in Henan Province. These instructors distributed the survey link to nursing interns who met the inclusion criteria. Subsequently, those participants were encouraged to forward the link to other eligible nursing interns across the country. To ensure quality control in the snowball sampling process, the following measures were implemented: (1) initial participants were provided with standardized screening criteria and a brief training video outlining the inclusion/exclusion criteria; (2) all forwarded links were tracked, and participants were required to confirm their eligibility through a screening question at the beginning of the survey; (3) IP addresses were monitored to identify potential duplicate submissions; and (4) a contact email was provided for participants to verify their eligibility if any questions arose.

The inclusion criteria include nursing students who had completed nursing courses, engaged in clinical internship work, had not yet obtained a nursing qualification certificate, were willing to participate in research. Exclusion criteria include those participants who are unable to continue participating in the study, or incomplete questionnaires. This study defines an invalid questionnaire as one where the same option is provided for each item.

According to Kendall’s (Bonett and Wright, 2000) method, the estimated sample size required for the study is 5–10 times the number of selected variable dimensions, as applied in a recent study (Liu K. et al., 2025). The variables required for this study include 36 items (11 demographic items + 10 self-efficacy items + 4 feedback-seeking items + 6 transition shock items + 5 professional identity items). According to Kendall’s (McGinness et al., 2020) method, the recommended sample size is 5–10 times the number of items, yielding a range of 180–360 participants. Accounting for a 20% invalid questionnaire rate, the final sample size should be between 216 and 432 participants. The actual sample of 450 participants exceeded this requirement, providing adequate statistical power.

### 2.3 Instruments

#### 2.3.1 General information questionnaire

Based on previous relevant studies (Zhang et al., 2023; Zhao et al., 2024), this study includes 11 variables of socio-demographic characteristics: gender, age, reason for choosing nursing major, whether there are nurses in the family, physical health status, whether one likes the nursing major, whether to serve as class leader, whether to be an only child, father’s education level, mother’s education level, and family’s per capita monthly income.

#### 2.3.2 Transition shock scale for undergraduate nursing students (UNSTS)

This scale was developed by Kim and Shin (2019), and localized by Weng et al. (2025). It has been used to assess the degree of transition Shock on nursing interns, including conflicts between theory and practice (3 items), excessive workload during internships (3 items), lack of social support (2 items), tense interpersonal relationships (3 items), confusion about the value of the nursing profession (5 items), and dissonance between clinical internships and personal life (2 items) across 6 dimensions, totaling 18 items (Li et al., 2024). A 4-point Likert scale (1 = strongly disagree, 4 = strongly agree) is used, the total score ranges from 18 to 72 with higher scores indicating a more severe perception of the transformational impact on the individual. The Cronbach’s α coefficient of the Chinese scale is 0.912, and the content validity is 0.987, indicating good reliability and validity (Weng et al., 2025). The Cronbach’s α coefficient of this scale in the present study is 0.79.

#### 2.3.3 General Self-Efficacy Scale (GSES)

The scale was developed by Schwarzer et al. (1997) and was used to assess self-efficacy in nursing students and nurses (Berdida et al., 2023; Li et al., 2024), and translated into Chinese by Zhang and Schwarzer (1995). It is used to assess students’ general self-efficacy and consists of a single dimension with 10 items (Ma and Ma, 2025). A 4-point Likert scale ranging from 1 (completely incorrect) to 4 (completely correct) is used, with a total score between 10 and 40 points; the higher the score, the higher the individual’s sense of self-efficacy. The internal consistency coefficient for the scale was 0.89. The results of the confirmatory factor analysis suggest that the scale possesses strong structural validity, as evidence by the following indices: χ^2^/df = 2.82, RMSEA = 0.055, NFI = 0.961, GFI = 0.970, CFI = 0.974 (Liu et al., 2024). The Cronbach’s α coefficient of this scale in the present study is 0.95.

#### 2.3.4 Feedback-Seeking Behavior Scale (FSBS)

This scale was developed by McGinness et al. (2020), translated and revised by Gong and Li (2019) to investigate employees’ feedback-seeking behavior. In a Chinese study, the Cronbach’s α coefficient of the scale is 0.890 (Lian and Zhang, 2020; Xiong et al., 2026). To adapt to the needs of nursing students, this study adjusted the scale, which includes four dimensions: seeking observational feedback from mentors/teachers (2 items), seeking inquiry-based feedback from mentors/teachers (2 items), seeking observational feedback from colleagues/classmates (3 items), and seeking inquiry-based feedback from colleagues/classmates (4 items), totaling 11 items. A 7-point Likert scale ranging from 1 (strongly disagree) to 7 (strongly agree) is used, the total score ranges from 11 to 77 points, with a higher score indicating better feedback-seeking behavior. The Cronbach’s α coefficient of this scale in the present study is 0.98.

#### 2.3.5 Professional Identity Questionnaire for Nurse Students (PIQNS)

This scale was developed by Hao (2011), based on the hybrid model for concept development of nurse professional identity constructed by Ohlén and Segesten (1998). It is widely used to assess nursing students professional identity in China (Ding et al., 2024). It includes 5 dimensions with a total of 17 items: occupational self-concept (6 items), retention benefits and resignation risks (4 items), social support and self-reflection (3 items), career choice autonomy (2 items), and social persuasion (2 items). A 5-point Likert scale is used, ranging from 1 (very inconsistent) to 5 (very consistent), with item 12 scored in reverse. The total score ranges from 17 to 85, with higher scores indicating a stronger professional identity. The Cronbach’s α coefficient of the scale is 0.827, the confirmatory factor analysis shows *X*^2^/df = 1.638, RMSEA = 0.0576, indicating that the questionnaire has good reliability and validity (Wang M. et al., 2024). The Cronbach’s α coefficient of this scale in the present study is 0.94.

### 2.4 Data collection

We conducted an online survey using the Questionnaire Star survey platform.^1^ First, the three authors of this study acted as investigators for data collection, and they received standardized training to better understand the purpose of the research and the important considerations for accurately distributing the questionnaires. Secondly, the investigator contacted the overall instructor at the hospital and obtained verbal consent. The investigator added the instructor on WeChat and joined the work group. Then, the investigator randomly selected samples from the WeChat group that met the inclusion criteria as research subjects, sharing with them the research content, data privacy, and protection related to this study. Participants were asked to scan a QR code to fill an online questionnaire. Allow participating researchers to recommend subjects that meet the inclusion criteria for this study. To avoid information bias, all questions are set as mandatory, so there are no missing values among the selected participants, and each IP address or phone number can only be submitted once. As of March 19, 2025, a total of 451 nursing interns completed the questionnaire for this study. After excluding 1 invalid questionnaire (where all selected options were the same), there were 450 valid questionnaires, meeting the required sample size.

### 2.5 Data analysis

Data analysis was conducted using R software (4.4.1). To assess the potential for common method bias, Harman’s single-factor test was conducted using exploratory factor analysis. All items from the four scales were entered into a principal component analysis. Additionally, variance inflation factor (VIF) values were calculated to assess multicollinearity among the predictor variables, with VIF values > 10 indicating concerning levels of collinearity. The Shapiro-Wilk test was used to test the normality of continuous data. If the continuous data follow a normal distribution, they are described by the mean and standard deviation (SD). If the continuous data do not follow a normal distribution, they are described using the median and interquartile range (IQR). Categorical variables are described by frequency and percentage. Pearson correlation analysis was used to examine whether there is a correlation between self-efficacy, feedback-seeking behavior, transition shock, and professional identity. Independent sample *t*-tests and one-way ANOVA were used to assess the association between vocational identity and categorical variables in social demographic characteristics. In addition, multiple linear regression analysis was conducted to determine the relationship between transition shock, feedback-seeking behavior, and professional identity, while controlling for confounding covariates. We used the R package “mediation” to test the mediation model and employed the Bootstrap method to examine the mediating effect of feedback-seeking behavior between transition shocks and professional identity, setting 1,000 bootstrap samples to calculate the 95% CI of the indirect effect. In addition, the R package “lavaan” was used to further examine the moderating effect of self-efficacy between transition shock and feedback-seeking behavior, and to calculate the impact of transition shock on feedback-seeking behavior at three different levels: mean - SD, mean, and mean + SD, as well as the mediating role of feedback-seeking behavior in the relationship between transition shock and professional identity. A two-sided *p*-value < 0.05 is considered statistically significant.

## 3 Results

### 3.1 Sample characteristics

A total of 451 clinical nursing interns participated and answered the questionnaire. According to the source of the IP addresses, there were 166 from Hebei (36.81%), 63 from Henan (13.97%), 63 from Jiangsu (13.97%), 32 from Zhejiang (7.10%), 16 from Jiangxi (3.55%), 15 from Shandong (3.33%), 14 from Guangdong (3.10%), 13 from Anhui (2.88%), 12 from Hubei (2.66%), 10 from Sichuan (2.22%), 9 from Fujian (2.00%), 8 from Liaoning (1.77%), 5 from Guangxi (1.11%), 5 from Beijing (1.11%), 5 from Hunan (1.11%), 5 from Shaanxi (1.11%), 2 from Xinjiang (0.44%), 2 from Gansu (0.44%), 2 from Qinghai (0.44%), 1 from Shanxi (0.22%), 1 from Jilin (0.22%), 1 from Shanghai (0.22%), and 1 from Tianjin (0.22%). One invalid questionnaire was excluded (where all selected options were the same), leaving 450 valid questionnaires for a validity rate of 99.8%. The data shows that there were 163 males (36.2%) and 287 females (63.8%). The average age of the study subjects was 23.13 ± 3.19 years, and the average internship duration was 9.20 ± 0.79 months. The descriptive statistical results of 450 clinical nursing interns are shown in Table 1.

**TABLE 1:** Participants’ demographics and differences in professional identity (*N* = 450).

Characteristics	Categories	No.(%)	Professional identity		
			Mean ± SD	*t/F*	*p*
Gender	Male	163 (36.2)	38.5 ± 13.0	−6.53	< 0.001
	Female	287 (63.8)	47.3 ± 15.1		
Major selection reason	Self-interest	118 (26.2)	41.2 ± 16.5	11.19	<0.001
	Recommended by family/friends	200 (44.4)	47.8 ± 14.6		
	Others	132 (29.3)	41.2 ± 12.9		
Whether family has a nurse	Yes	181 (40.2)	42.9 ± 14.4	−1.45	0.149
	No	269 (59.8)	44.9 ± 15.3		
Self-report health	Health	243 (54.0)	49.2 ± 16.2	35.95	<0.001
	Average	158 (35.1)	39.1 ± 11.3		
	Unhealthy	49 (10.9)	35.2 ± 8.54		
Like nursing major	Like	174 (38.7)	42.9 ± 17.0	41.15	<0.001
	Moderate	167 (37.1)	50.9 ± 12.7		
	Dislike	109 (24.2)	35.7 ± 8.90		
Class leader	Yes	167 (37.1)	40.3 ± 14.0	−4.36	<0.001
	No	283 (62.9)	46.4 ± 15.1		
Only child	Yes	143 (29.8)	38.4 ± 12.7	−5.83	<0.001
	No	316 (70.2)	46.5 ± 15.3		
Father’s educational level	Junior high school or below	186 (41.3)	47.1 ± 15.6	6.66	<0.002
	High school or technical secondary school	133 (29.6)	42.8 ± 14.0		
	College or above	131 (29.1)	41.3 ± 14.4		
Mother’s educational level	Junior high school or below	212 (47.1)	47.4 ± 15.4	11.04	<0.001
	High school or technical secondary school	130 (28.9)	42.5 ± 14.4		
	College or above	108 (24.0)	39.6 ± 13.6		
Average monthly income per family member	Less than 3,000 yuan	120 (26.7)	44.0 ± 16.1	2.30	0.102
	3,000∼5,000 yuan	188 (41.8)	45.7 ± 14.7		
	More than 5,000 yuan	142 (31.6)	42.1 ± 14.4		

### 3.2 Univariate analysis

The results of this study show that the professional identity of females are higher than those of males (47.3 ± 15.1 vs. 38.5 ± 13.0, *p* < 0.001), the professional identity of students who do not serve as class leaders are higher than those of students who do serve as class leaders (46.4 ± 15.1 vs. 35.7 ± 8.90, *p* < 0.001), and the professional identity of non-only child are higher than those of only child (46.5 ± 15.3 vs. 38.4 ± 12.7, *p* < 0.001). In addition, factors such as the reason for choosing the nursing major (*p* < 0.001), self-report health (*p* < 0.001), whether one likes the nursing major (*p* < 0.001), father’s education level (*p* < 0.002), and mother’s education level (*p* < 0.001) are associated with the professional identity of nursing interns (see Table 1).

### 3.3 Common method bias and multicollinearity assessment

Harman’s single-factor test revealed that the first factor accounted for 20.1% of the total variance, which is below the recommended threshold of 40%, suggesting that common method bias was not a significant concern in this study. Additionally, VIF values ranged from 1.05 to 2.17, all below the threshold of 10, indicating no significant multicollinearity among the predictor variables (Supplementary Table 1).

### 3.4 Correlation analysis

The results of the correlation analysis show that transition shock is negatively correlated with feedback-seeking behavior (*r* = −0.711), negatively correlated with self-efficacy (*r* = −0.651), and negatively correlated with professional identity (*r* = −0.754). Feedback-seeking behavior is positively correlated with self-efficacy (*r* = 0.831), positively correlated with professional identity (*r* = 0.862), and self-efficacy is positively correlated with professional identity (*r* = 0.807) (all *p* < 0.001, Table 2).

**TABLE 2:** Pearson correlation analysis.

r (95% CI)*	Mean ± SD	Transition shock	Feedback-seeking behavior	Self-efficacy	Professional identity
Transition shock	55.10 ± 11.20	1	1	1	1
Feedback-seeking behavior	35.98 ± 21.79	−0.711 (−0.754, −0.662)			
Self-efficacy	20.67 ± 7.25	−0.651 (−0.701, −0.594)	0.831 (0.800, 0.858)		
Professional identity	44.11 ± 15:00	−0.754 (−0.791, −0.711)	0.862 (0.837, 0.885)	0.807 (0.772, 0.837)	

*The above all *p* < 0.001.

### 3.5 Multivariate linear regression analysis

In Model 1, professional identity was regressed on transition shock. Transition shock demonstrated a significant negative association with professional identity (β = −0.841, *p* < 0.001), indicating that higher transition shock scores corresponded to lower professional identity scores. In Model 2, feedback-seeking behavior was regressed on transition shock, revealing a significant negative association (β = −1.058, *p* < 0.001). In Model 3, professional identity was simultaneously regressed on transition shock and feedback-seeking behavior. The results showed that transition shock remained negatively associated with professional identity (β = −0.368, *p* < 0.001), whereas feedback-seeking behavior was positively associated with professional identity (β = 0.447, *p* < 0.001). All regression coefficients are presented in Table 3.

**TABLE 3:** Multiple linear regression analysis.

Model variables	β	SE	*t*	*P*-value
Model 1 (outcome: Professional identity)				
Transition shock	−0.841	0.043	−19.413	<0.001
Model 2 (outcome: Feedback-seeking behavior)				
Transition shock	−1.058	0.064	−16.469	<0.001
Model 3 (outcome: Professional identity)				
Transition shock	−0.368	0.041	−8.894	<0.001
Feedback-seeking behavior	0.447	0.024	18.461	<0.001

### 3.6 Mediation model construction

**FIGURE 2:**
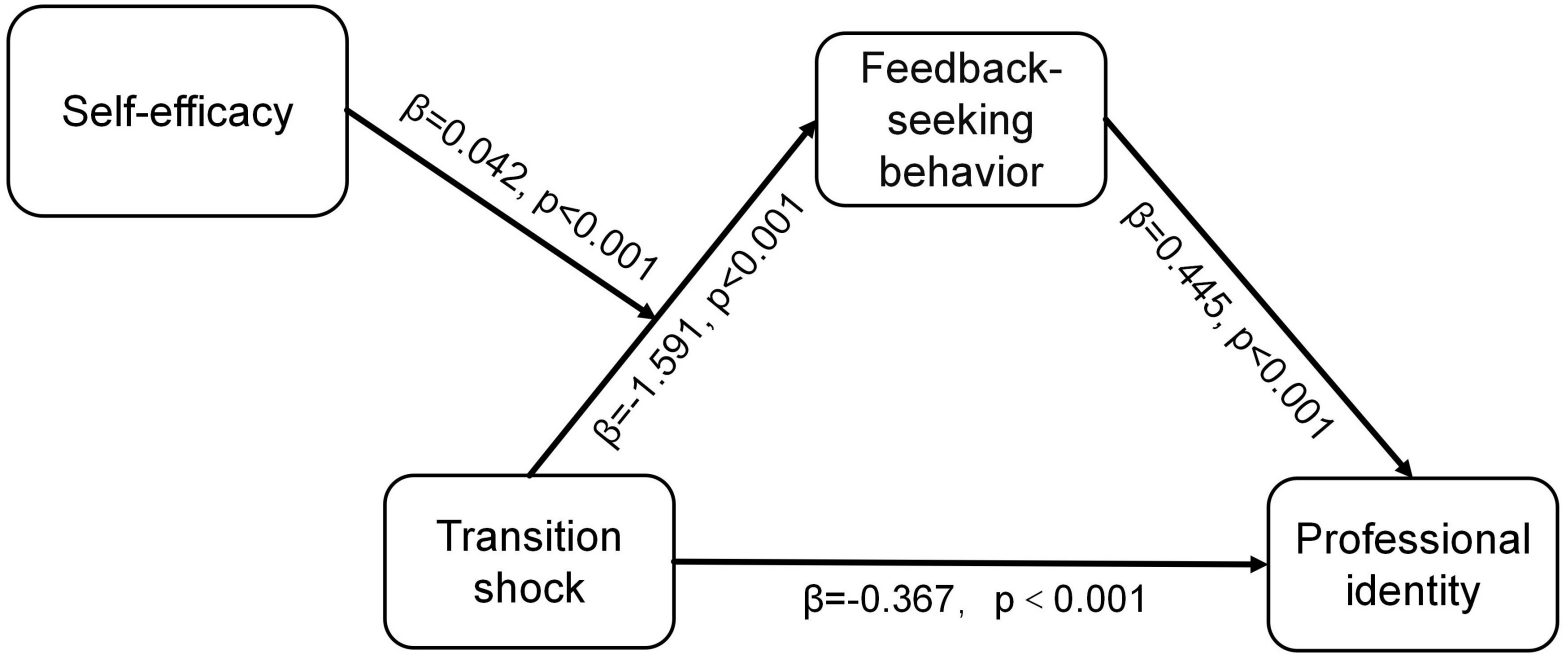
Moderated mediation analysis results.

After controlling for covariates, feedback-seeking behavior has a mediating effect on transition shock and professional identity, as shown in the complete model results in Figure 2 and Table 4. Overall, the total effect of transition shock on professional identity is significant (β = −0.841, *p* < 0.001). Transition shock has a significant direct effect on professional identity (β = −0.368, *P* < 0.001), indicating that higher transition shock corresponds to lower professional identity. Feedback-seeking behavior can partially mediate the relationship between transition shock and professional identity (indirect effect = −0.473, 95% CI: −0.557 to −0.391). The proportion of the total effect is 56.3%.

**TABLE 4:** The results of the mediation effect analysis.

Variables	β	95%CI		*p*-value
		Lower	Upper	
Indirect effect	−0.473	−0.557	−0.391	<0.001
Direct effect	−0.368	−0.494	−0.248	<0.001
Total effect	−0.841	−0.956	−0.704	<0.001
Mediated proportion	0.563	0.464	0.675	<0.001

### 3.7 Moderated mediation model construction

Regression analysis suggested that the interaction term of self-efficacy and transition shock is significantly associated with feedback-seeking behavior (β = 0.042, *P* < 0.001), as shown in Figure 2.

At low levels of self-efficacy, the negative association between transition shock and feedback-seeking behavior was stronger (β = −1.634); at moderate levels of self-efficacy, this association was somewhat weaker (β = −1.591); and at high levels of self-efficacy, the association was weakest (β = −1.549). These findings indicate that the negative association between transition shock and feedback-seeking behavior diminished as self-efficacy increased (all *p* < 0.001).

The indirect effect of feedback-seeking behavior on the relationship between transition shock and professional identity varied across levels of self-efficacy (Table 5). At low self-efficacy, the indirect effect was −0.726 (95% CI: −0.872 to −0.581), accounting for 86.3% of the total effect. At moderate self-efficacy, the indirect effect was −0.707 (95% CI: −0.848 to −0.566), explaining 84.1% of the total effect. At high self-efficacy, the indirect effect was −0.689 (95% CI: −0.825 to −0.552), representing 81.9% of the total effect.

**TABLE 5:** The effect of transition shock on feedback-seeking behavior at different levels of self-efficacy and the differences in indirect effect values.

Levels	Estimate	SE	*z*-value	*p*	95%CI		Indirect effect/
					Lower	Upper	total effect
Effect of transition shock on feedback seeking behavior at different self-efficacy level							
Mean – SD	−1.634	0.145	−11.277	<0.001	−1.918	−1.350	–
Mean	−1.591	0.140	−11.346	<0.001	−1.866	−1.316	–
Mean + SD	−1.549	0.136	−11.417	<0.001	−1.815	−1.283	–
Indirect effect at different self-efficacy level							
Mean - SD	−0.726	0.074	−9.787	<0.001	−0.872	−0.581	0.863
Mean	−0.707	0.072	−9.832	<0.001	−0.848	−0.566	0.841
Mean + SD	−0.689	0.070	−9.878	<0.001	−0.825	−0.552	0.819

## 4 Discussion

This study firstly explored the relationship between transition shock of nursing interns and their professional identity, especially the roles of self-efficacy and feedback-seeking behavior in this process. The research results indicated that transition shock is negatively correlated with nursing interns’ professional identity, with feedback-seeking behavior as a mediating factor, self-efficacy plays a moderating role, buffering the negative association between transition shock and professional identity.

In this study, the professional identity score of nursing interns was (44.11 ± 15.00), which is at a low level. It is lower than the findings of Wang M. et al. (2024) which reported a score of (58.37 ± 10.69) for 365 nursing undergraduates from 13 universities in China, and also lower than the results of a cross-sectional study conducted by Zhang et al. (2024), which reported a score of (60.77 ± 12.18) for 1,050 nursing interns from 26 teaching hospitals in Sichuan Province, China.

The development of professional identity among nursing interns is a dynamic process shaped by multiple factors, and significant differences exist across various sociodemographic characteristics (Ding et al., 2024; Mao et al., 2021). This study demonstrated that females’ professional identity is higher than males’, consistent with the findings of Woo and Park (2017; Xu et al. (2025). This may be attributed to the stereotype that nursing is a female profession (Metzger et al., 2020; Prosen, 2022). The results of this study showed that the professional identity of a non-only child is higher than that of an only child. A possible reason is that, influenced by China’s family planning policy, only children generally receive more care during their growth, while the dual characteristics of nursing work, which involves both physical and mental labor, contrast sharply with the long-term living environment of only children, leading to significantly lower professional identity compared to non-only children (Peng et al., 2024; Yang et al., 2025). Parents’ educational level is associated with nursing interns’ professional identity. A possible reason is confusion about career choices and development paths during the internship stage; at this time, parents’ cultural literacy, life experiences, and social resources can directly shape nursing students’ professional identity through career advice (Chen et al., 2025; Gao et al., 2021). The reasons for choosing the nursing major may also correlate with the professional identity of nursing interns, consistent with the findings of Shi et al. (2025), who found that, compared with nursing students assigned to the major, those who autonomously chose the nursing major have a higher level of professional identity. In addition, this study demonstrated that students’ professional identity is higher among those who do not serve as class leaders than among those who do. Factors such as physical health status and whether students like the nursing profession may also connect with the professional identity of nursing students. Therefore, it is recommended that school and hospital administrators develop targeted personalized training strategies based on the demographic characteristics of nursing students to effectively enhance their sense of professional identity.

This study demonstrated that transition shock negatively correlates with the professional identity of nursing interns (β = −0.368, *p* < 0.001), a findings similar to that of Wang M. et al. (2024) among 365 nursing undergraduates from 13 universities in China. Possible reasons are that nursing interns are in their graduation year, facing the dual pressures of continuing education or employment (Tang et al., 2025), which can easily lead to experiences of anxiety, confusion, and helplessness during this transitional period (Ko and Kim, 2022). This may be directly associated with their role positioning and core values, leading to negative career exploration and indecision and a lower sense of professional identity (Abdelaziz et al., 2025; Labrague and De Los Santos, 2020).

This study found that feedback-seeking behavior is positively correlated with the professional identity of nursing interns, indicating that good feedback-seeking behavior enhances the professional identity of nursing interns, which is consistent with previous research findings (Ding et al., 2024). Based on social cognitive theory, feedback-seeking behavior promotes individuals’ self-regulation (Hwang, 2025; Smoktunowicz et al., 2017). The stronger the feedback-seeking behavior of nursing interns, the better they are at self-regulation, actively exploring their environment, and expressing and evaluating, thus achieving two-way feedback between teachers and students, as well as among nursing students (Ramani et al., 2019; Xiong et al., 2026). This way, they can better solve the problems they encounter, continuously build good relationships with classmates, and gain satisfaction in learning and work, which in turn leads to a strong sense of professional identity (Li et al., 2021; Yao and Wu, 2025).

This study demonstrated that feedback-seeking behavior plays a mediating role between transition shock and professional identity, meaning that transition shock may connect with professional identity through feedback-seeking behavior, which validates Hypothesis 3. This study suggested that transition shock has a negative association with the feedback-seeking behavior of nursing interns. The higher the transition shock experienced by nursing interns, the lower their feedback-seeking behavior, which is consistent with previous research findings (Ma et al., 2022; Zhang et al., 2023). A possible reason is that nursing interns may face difficulties during environmental transitions due to inadequate preparation and a lack of professional skills, making them more prone to negative professional evaluations, which in turn reduces their initiative to seek feedback (Wenxia et al., 2022). When the level of transition shock is high, nursing interns often find themselves in a highly sensitive state, prone to psychological distress, showing signs of negative burnout, and lacking the internal motivation to engage in feedback-seeking behavior (Eckerson, 2018; Li et al., 2025).

An important finding of this study is that self-efficacy moderated the relationship between transition shock and feedback-seeking behavior, which validates hypothesis 4. Specifically, for interns with high self-efficacy, the negative association between transition shock and feedback-seeking behavior is weaker while for interns with low self-efficacy, this negative impact is stronger. Previous studies have also reported similar results (Rafiei et al., 2024). Self-efficacy is the most influential factor in promoting innovative behavior (Ma and Ma, 2025). According to the “stress-elasticity” framework, self-efficacy is an important influencing factor of individual attitudes and behaviors when coping with stress and challenges (Berdida et al., 2023; Li et al., 2024; Schwarzer et al., 1997). When facing stress from role transitions, nursing interns with high self-efficacy can still actively mobilize their own resources, seek effective coping behaviors, and take the initiative to master nursing knowledge and skills, making it easier for them to receive positive feedback during their internships (Wang M. et al., 2024). However, nursing interns with low self-efficacy cannot engage in proactive self-regulation when faced with high stress; instead, they experience negative emotions such as resistance and fatigue, which may make them more likely to engage in avoidant behaviors.

Self-efficacy and feedback-seeking behavior are important associated factors of the professional identity among nursing students. First, nursing educators and hospital managers should focus on enhancing nursing interns’ self-efficacy and feedback-seeking behaviors by providing them with multi-level scientific training strategies, such as increasing practical teaching, case-based teaching, flipped classrooms (Chao and Liao, 2025) and immersive teaching (Chao et al., 2025) among other diverse training courses (Butte et al., 2025). This approach will strengthen nursing students’ sense of participation and achievement, boost their learning motivation, ensure deeper learning, improve their adaptability in the face of adversity, and facilitate role transformation and adaptation (Yu et al., 2024). Secondly, clinical instructors should focus on the emotional management of nursing interns, attempting to incorporate ideological and political education into clinical practice, enabling nursing students to gain positive emotional guidance during practice, better cope with work-related setbacks, reduce their feelings of depression, and enhance their resilience (Liu Z. et al., 2025). Finally, nursing interns should continue to strengthen their professional theoretical knowledge and practical skills through clinical practice, continually accumulating experience, reinforcing professional confidence, and developing the ability to independently cope with setbacks in their work. This will help them better handle the sources of stress during their internship, cultivate professional values, and construct professional identity (Wu et al., 2024).

## 5 Research limitations and future directions

This study has several limitations. First, the research design of this study adopted a cross-sectional design, which may make it difficult to strictly infer causal relationships between variables (Rindfleisch et al., 2008; Xin et al., 2024). Future research could adopt a longitudinal design or daily diary method to more accurately capture changes in variables over time and the causal order. Secondly, this study’s measurement method relied on self-report questionnaires, which may be subject to common method bias (Heckman, 1977; Zhu et al., 2024). Future research could employ multi-source data (such as colleagues/mentors evaluating feedback-seeking behaviors), behavioral observations, or objective indicators for complementary validation. Additionally, the sample in this study may have come from certain regions or a few hospitals, limiting the generalizability of the conclusions, and there may be some errors in the representativeness of the sample. Future research should expand the sampling range and scale, conduct multicenter studies, and explore model differences in different cultural contexts. In terms of model construction, this study may have omitted other important variables (such as social support, mentoring style, organizational climate, etc.) (MacKinnon, 2008; Wang T. T. et al., 2024). Future research could incorporate these as moderating variables or competitive mediating variables into the model to build a more comprehensive explanatory network. Fourth, this study employed a snowball sampling method, which may introduce selection bias and limit the generalizability of findings (Goodman, 1961; Liu et al., 2024). While efforts were made to ensure quality control through standardized screening procedures, the reliance on participant referrals may have led to a sample that is not fully representative of the broader population of nursing interns across China. Notably, the sample was predominantly from Hebei Province (36.81%), with limited representation from several other provinces, which may affect the external validity of our findings. Future research should employ probability sampling methods, such as stratified random sampling across multiple geographic regions, to enhance representativeness. The term “clinical nursing interns in China” should be interpreted with caution, as the current sample may not adequately represent nursing interns in regions with limited participation.

## 6 Conclusion

Transition shock demonstrated a negative correlation with professional identity in this sample of nursing interns, with feedback-seeking behavior acting as a mediating factor, while self-efficacy plays a moderating role. Therefore, nursing managers should adopt comprehensive and in-depth intervention measures to improve nursing interns’ self-efficacy and feedback-seeking behavior, address the negative association between transition shock and professional identity, enhance the professional identity of nursing interns, and promote the stable development of the nursing workforce.

## Data Availability

The raw data supporting the conclusions of this article will be made available by the authors, without undue reservation.

## Footnotes

1
https://www.wjx.cn/
