# Supplementary material for: The relationship between transition shock and professional identity of nursing interns in China: a moderated mediation effect analysis
Source: Front Psychol. 2026 Aug 4;17:1886969. doi: 10.3389/fpsyg.2026.1886969 (PMC13481449; doi:10.3389/fpsyg.2026.1886969)
Supplement: Supplementary file 1 [file Table_1.docx]

| Supplementary Table 1 Multicollinearity Test Results | |
| --- | --- |
| **Variable** | **VIF** |
| Transition shock | 1.458 |
| Self-efficacy | 1.856 |
| Feedback-seeking behavior | 2.170 |
| Gender | 1.078 |
| Major selection reason | 1.050 |
| Self-report health | 1.063 |
| Like nursing major | 1.093 |
| Class leader | 1.052 |
| Only child | 1.090 |
| Father's Educational level | 1.072 |
| Mother's Educational level | 1.078 |
